# Circular RNA profiling of the rice photo-thermosensitive genic male sterile line Wuxiang S reveals circRNA involved in the fertility transition

**DOI:** 10.1186/s12870-019-1944-2

**Published:** 2019-08-05

**Authors:** Ying Wang, Zeyang Xiong, Qian Li, Yueyang Sun, Jing Jin, Hao Chen, Yu Zou, Xingguo Huang, Yi Ding

**Affiliations:** 1 State Key Laboratory of Hybrid Rice, Department of Genetics, College of Life Sciences, Wuhan University, Wuhan, 430072 China; 2 WuhanWuda Tianyuau Bio-Tech Co., LTD, Wuhan, China

**Keywords:** circRNA, PTGMS rice, Fertility transition, RNA-sequencing, miRNA sponge

## Abstract

**Background:**

Circular RNAs (circRNAs) are known to play an important role in the regulation of gene expression in eukaryotes. Photo-thermosensitive genic male sterile (PTGMS) is a very important germplasm resource in two-line hybrid rice breeding. Although many circRNAs have been identified in rice (*Oryza sativa* L*.*), little is known about the biological roles of circRNAs in the fertility transition of the PTGMS rice line.

**Results:**

In the present study, RNA-sequencing libraries were constructed from the young panicles of the Wuxiang S sterile line rice (WXS (S)) and its fertile line rice (WXS (F)) at three development stages with three biological replicates. A total of 9994 circRNAs were obtained in WXS rice based on high-throughput strand-specific RNA sequencing and bioinformatic approaches, of which 5305 were known circRNAs and 4689 were novel in rice. And 14 of 16 randomly selected circRNAs were experimentally validated with divergent primers. Our results showed that 186 circRNAs were significantly differentially expressed in WXS (F) compared with WXS (S), of which 97, 87 and 60 circRNAs were differentially expressed at the pollen mother cell (PMC) formation stage (P2), the meiosis stage (P3) and the microspore formation stage (P4), respectively. Fertility specific expression patterns of eight circRNAs were analysis by qRT-PCR. Gene ontology (GO) and KEGG pathway analysis of the parental genes of differentially expressed circRNAs (DECs) revealed that they mainly participated in various biological processes such as development, response to stimulation, hormonal regulation, and reproduction. Furthermore, 15 DECs were found to act as putative miRNA sponges to involved in fertility transition in PTGMS rice line.

**Conclusion:**

In the present study, the abundance and characteristics of circRNAs were investigated in the PTGMS rice line using bioinformatic approaches. Moreover, the expression patterns of circRNAs were different between WXS (F) and WXS (S). Our findings primarily revealed that circRNAs might be endogenous noncoding regulators of flower and pollen development, and were involved in the fertility transition in the PTGMS rice line, and guide the production and application of two-line hybrid rice.

**Electronic supplementary material:**

The online version of this article (10.1186/s12870-019-1944-2) contains supplementary material, which is available to authorized users.

## Background

Circular RNAs (circRNAs) comprise a pervasive and vital class of noncoding RNAs characterized by a single-stranded covalently closed ring structure [1, 2]. They are mainly derived from a back-splicing event in which an upstream 5′ splice acceptor is linked to a downstream 3′ splice donor [3–5]. Based on the origin of their components, circRNAs can be divided into exonic circRNAs, intronic circRNAs and intergenic circRNAs [6, 7]. In the 1970s, Sanger et al. [8] provided the first evidence for the presence of circRNAs in some plant viroids. Despite their detection for decades, circRNAs used to be considered rare in eukaryotic cells or disregarded as splicing errors or reverse transcription (RT)-PCR artifacts. Until recently, due to the advance of high-throughput sequencing and high-efficiency bioinformatics methods [9–12], understanding of the widespread and diverse circRNAs has improved substantially in a variety of organisms [13–17].

Recent studies in humans and animals have shown that the expression patterns of circRNAs are cell-type, developmental-stage or tissue-specific, implying their crucial regulatory roles in a various of biological processes [2, 13, 18, 19]. Increasing evidence has demonstrated that circRNAs can function as miRNA sponges [20, 21] and can regulate the transcription and splicing of parental genes [7, 22, 23]; they even can be translated into polypeptides or proteins [24, 25]. In contrast, the biological roles of circRNAs in plants are poorly understood, as the first exploration of plant circRNA was carried out in *Arabidopsis thalianain* in 2014 [15]. Recently, a growing number of circRNAs have been successfully detected in Arabidopsis [16, 26–30], rice [16, 17, 31], tomato [32–35], barely [36], maize [37–39], wheat [40], and soybean [41, 42], indicating that circRNAs are widely distributed in plants. These plant circRNAs have been found to be conserved and to have specific expression pattern, consistent with those in animals. However, the repetitive elements and reverse complementary sequences in the flanking regions of plant circRNAs are clearly reduced compared with animals [16]. Additionally, some circRNAs can act as positive or negative regulators of their parental genes in rice and Arabidopsis [16, 17]. Moreover, several reports have revealed that the majority of plant circRNAs exhibit differential expression patterns under various conditions. For example, 1583 circRNAs have been found to be uniquely expressed under heat stress in Arabidopsis [30], 160 circRNAs with differential expression have been identified in MIMV-infected maize and healthy maize [39], 62 circRNAs have been reported to be differentially expressed in response to dehydration stressin wheat [36], and 1009 circRNAs are differentially expressed between the soybean cytoplasmic male sterility (CMS) line and its maintainer line [42]. In addition, the molecular mechanisms of circRNAs in plants were revealed, in which an Arabidopsis circRNA generated from the *SEPALLATA3* (*SEP3*) gene reduces the expression of its parent gene by forming R-rings with the DNA locus [43]. Thereafter, by overexpressing constructs in Arabidopsis, a lariat-derived circRNA from At5g37720 regulates gene expression and affects plant development [44], and a circRNA (circGORK) from the GORK gene enhance plant ABA signaling and promote drought tolerance [45]. These findings indicate that plant circRNAs play a vital role in transcriptional and posttranscriptional regulatory processes, but nevertheless, additional analyses are still necessary to further clarify the function of circRNAs in plants.

Rice (*Oryza sativa* L*.*) is one of the most important crops worldwide and is an excellent model plant for monocotyledonous genomics research [46]. Over the past decades, hybrid rice breeding has dramatically increased the grain yield and contributed significantly to global food security, especially in China [47, 48]. Utilization of the photo-thermosensitive genic male sterile (PTGMS) line is an important approach to facilitate the development of two-line hybrid rice breeding [49, 50]. A major feature of PTGMS rice is that male sterility is caused by environmental factors such as photoperiod and temperature: the PTGMS line is male-sterile under high-temperatures and long-day conditions (restricted conditions), but it is converted to male-fertile under low-temperatures and short-day conditions (permissible conditions) [51, 52]. Thus, they can be used to propagate by self-pollination under permissible conditions and to produce hybrid seeds by outcrossing with restorer lines under restricted conditions. Moreover, the traits of PTGMS are controlled by nuclear recessive genes and any normal rice varieties can restore the fertility of F_1_ hybrids, provide a wide range of genetic resources for rice breeding, and produce hybrids with strong heterosis [49–52]. Thus far, a majority of genes that control photoperiod-sensitive genic male sterile (PGMS) or thermosensitive genic male sterile (TGMS) have been found in different rice lines [51], of which *pms3* and *tms5* are two widely used genes in rice breeding [53–55]. The *pms3* encodes a long noncoding RNA called LDMAR, which produces a 21-nucleotide small RNA. A single nucleotide polymorphism (SNP) between the wild-type and mutant leads to increased methylation in the putative promoter, which represses the transcription of LDMAR, and a loss-of-function of the small RNA under long-day conditions, eventually causing male sterility [53, 54]. The *tms5* encodes a RNase Z^S1^, which processes the mRNA of Ub_L40_, and a loss-of-function mutation in RNase Z^S1^ causes the mRNA of Ub_L40_ to clearly accumulate in the anthers, thus resulting male sterility under high-temperature conditions [55]. Although several effector genes of the PTGMS rice line have been fruitfully studied, most of the genetic regulatory mechanisms remain incompletely characterized.

To date, thousands of circRNAs have been identified in rice [16, 17]. However, no systematic study has been conducted to examine the potential role of circRNAs in the fertility transition of the PTGMS rice line. Wuxiang S (WXS) is a novel *indica* PTGMS rice line in the two-line system developed by our laboratory, characterized by low critical sterile temperature and strong combining ability. Our previous study has found that the sterility-fertility transition of WXS occurred when the ambient temperature was lower than 23.5 °C and short-day (< 14 h) conditions during anther development. We also found that some miRNAs could be involved in the regulation of pollen development in WXS [56]. In the present study, we performed genome-wide identification and characterization of circRNAs using high-throughput sequencing technology to investigate the expression profiles of circRNAs in the PTGMS rice line WXS and their potential roles in the fertility transition. Our results indicate that circRNAs might be involved in the regulation of anther and pollen development in the PTGMS rice line, and they provided a useful resource and new biological insights into the rice circRNAs.

## Result

### Identification of circRNAs in PTGMS rice WXS

To systematically identify the possible biological roles of cirRNAs in PTGMS rice that were involved in fertility transition, 18 RNA libraries from rice young panicles of WXS (S) and WXS (F) at the P2, P3 and P4 stages (SP2, SP3, SP4, FP2, FP3, and FP4) were constructed, with three biological replicates for each condition. These libraries were sequenced using an Illumina HiSeq Xten platform, and approximately 214.54 Gb clean reads were generated (Additional file 1: Table S1).

**Fig. 1:**
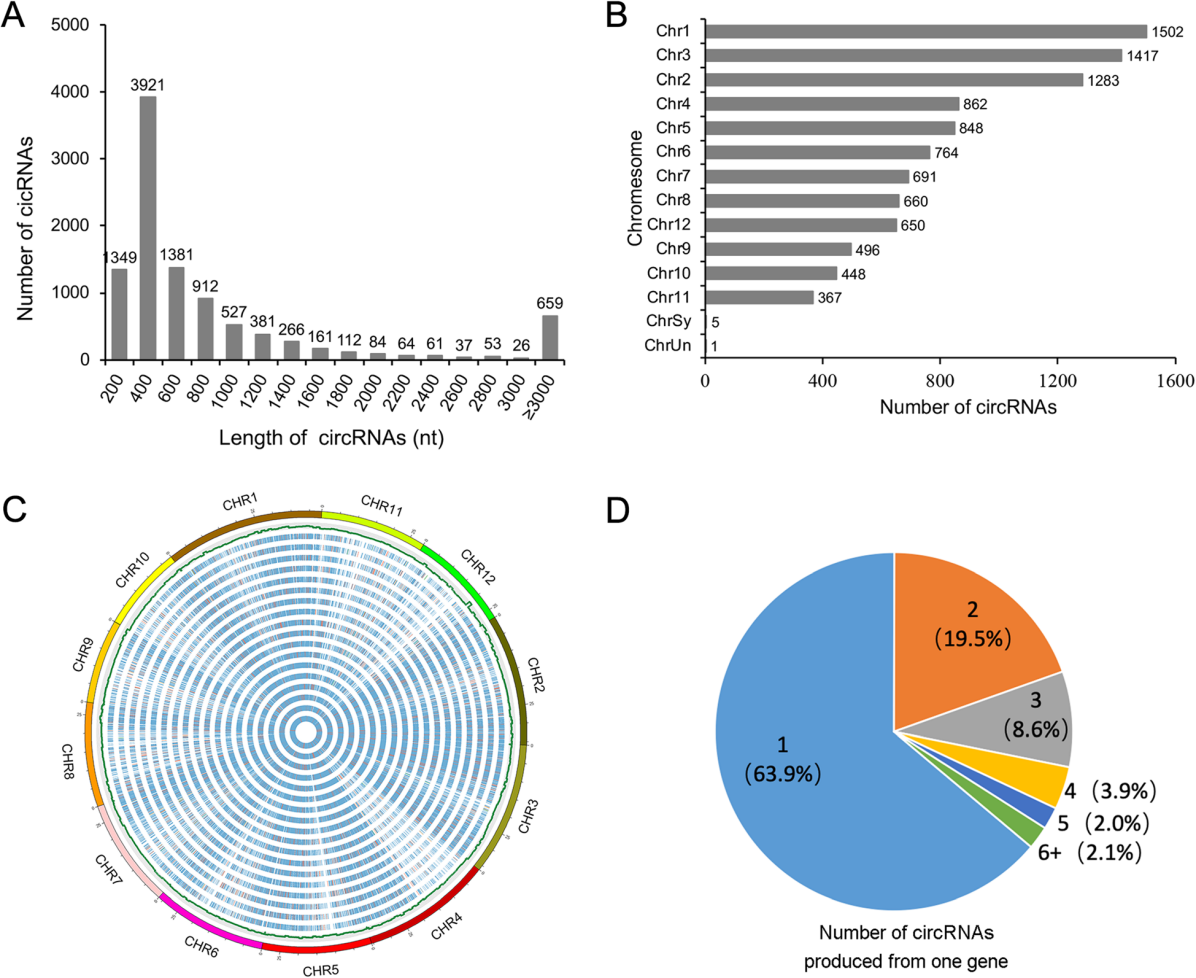
Characterization of circRNAs in PTGMS rice **a** The length distribution of identified circRNAs. **b** The histogram shows the number of circRNAs identified in each chromosome. **c** Circos plots shows the distribution of circRNA identified in rice (*Oryza sativa*) genome and their expression levels. The outmost histogram represents all rice chromosomes. The heat map shows the expression distribution of SP2–1, SP2–2, SP2–3, FP2–1, FP2–2, FP2–3, SP3–1, SP3–2, SP3–3, FP3–1, FP3–2, FP3–3, SP4–1, SP4–2, SP4–3, FP4–1, FP4–2, FP4–3 samples from inside to outside, respectively. **d** Number of circRNAs produced from one gene (8513 circRNAs from 4948 parental genes).

After careful screening and further bioinformatic analysis, a total of 9994 circular RNAs were obtained from all 18 samples in the study. In order to screen novel circRNAs, the back-splicing sequences of identified circRNAs were compared with those of rice circRNAs from PlantcircBase (http://ibi.zju.edu.cn/plantcircbase/) by BLASTN (E < 1e-5). Approximately 53% (5305) circRNAs in this study were known, and 47% (4689) circRNAs are novel in rice (Additional file 2: Table S2). Among these identified circRNAs in each sample, the proportion of exonic circRNA (68–79%) was significantly higher than intergenic RNA (19–28%) (Table 1), and the remaining intronic RNA (2–4%) was the least abundant, suggesting that circRNAs were mainly generated from exonic regions. We also observed the number of circRNAs in three different stages, and we found greater numbers in the P2 and P3 stages than in the P4 stage. It was indicated that the expression of circRNAs was development stage specific, which is consistent with previous reports that rice circRNAs often expressed specifically in different developmental stages [16]. Moreover, regarding the length range of the identified circRNAs, approximately 81% were shorter than 1 kb, while the mean length was 1603 (Fig. 1a). The chromosome distribution analysis showed that most of the circRNAs were generated from chromosome 1, followed by chromosomes 3 and 2 (Fig. 1b). Using the Circos program, the overall expression levels of circRNAs were found to be quite low (Fig. 1c). Additionally, a total of 8513 circRNAs were produced from 4948 parental genes. We observed that approximately 64% of the parental genes produced only one circRNA (Fig. 1d), although some parental genes could produce more than one circRNA.

### Experimental verification of the circRNAs candidates

**Fig. 2:**
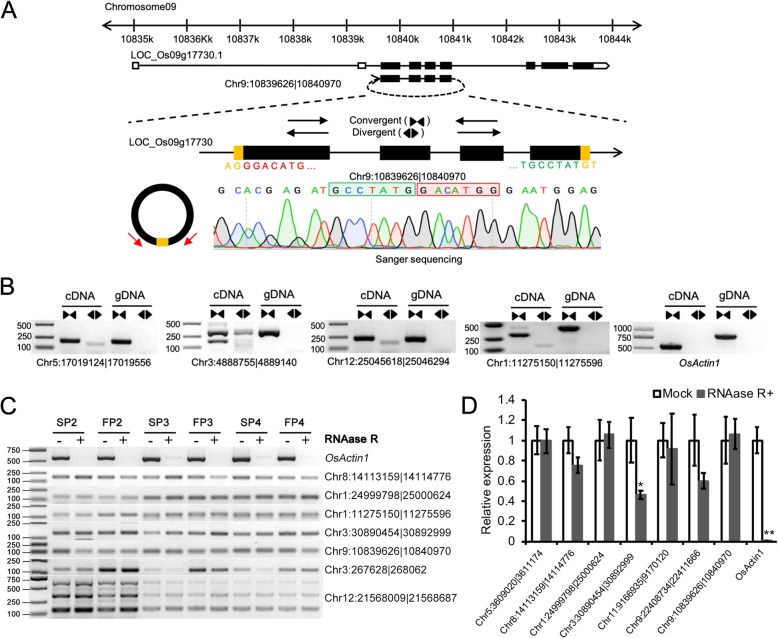
Experimental validated the stable expression of rice circRNAs **a** A rice circRNA (Chr9:10839626|10,840,970) exemplifies the validation strategy. According to the genomic loci, Chr9:10839626|10,840,970 derived from LOC_Os09g17730 gene. Red arrows represent divergent primers, which were designed to confirmed head-to-tail backsplicing by RT-PCR and sanger sequencing. **b** Divergent primers successfully amplify circRNAs in cDNA but not genomic DNA (gDNA). Convergent primers amplify circRNAs both in cDNA and gDNA. OsActin1 as a linear control. *n* = 3 replicates. **c** RT-PCR of RNase R (−) and RNase R (+) treated total RNA shows that circRNAs are stable transcripts. **d** qRT-PCR shows that seven circRNAs are more RNase R-resistant than linear mRNA. Data in (**d**) are the means ± standard deviation of three replicates. Asterisks indicate a significant difference as determined by Student’s *t*-test (*P < 0.05; **P < 0.01).

In the present study, we experimentally tested and verified the circRNA predictions in the PTGMS rice WXS. Since circRNAs were derived from head-to-tail splicing of RNA transcripts, convergent primers and divergent primers were designed and used to amplify the backsplice sites from total RNA, as well as genomic DNA. Unlike convergent primers, divergent primers could amplify the back-splicing junctions in cDNAs but not genomic DNAs. Additionally, the PCR bands amplified by divergent primers were further detected by sequencing (Fig. 2a). We successfully validated 14 of 16 randomly selected circRNAs (87.5%), including 11 (84.6%) exon circRNAs and three (100%) intragenic circRNAs, and our predictions presented a high degree of accuracy (Fig. 2b, Additional file 3: Figure S1).

To eliminate the interference of genomic rearrangements and potential PCR artifacts, half of the RNA samples were treated with the exonuclease RNAase R to degrade linear RNA molecules. The semiquantitative RT-PCR results showed that these circRNAs were stably expressed both with or without RNase R-treatment (RNase R (+) or RNase R (−)) samples, while the linear controls were almost completely degraded in RNase R (+) samples (Fig. 2c). Interestingly, three PCR-amplified bands were detected when PCR amplification was performed with divergent primers of circRNA Chr12:21568009|21,568,687, indicating alternative circularization events. We further detected the RNase R resistance of seven confirmed circRNAs by real-time quantitative RT-PCR (qRT-PCR). The results showed that these circRNAs were at least 40 times more resistant than *OsActin1* (Fig. 2d). We reasoned that circRNAs could represent a class of relatively stable transcriptional and posttranscriptional regulators in the PTGMS rice WXS.

### Analysis of expression patterns of circRNAs

**Fig. 3:**
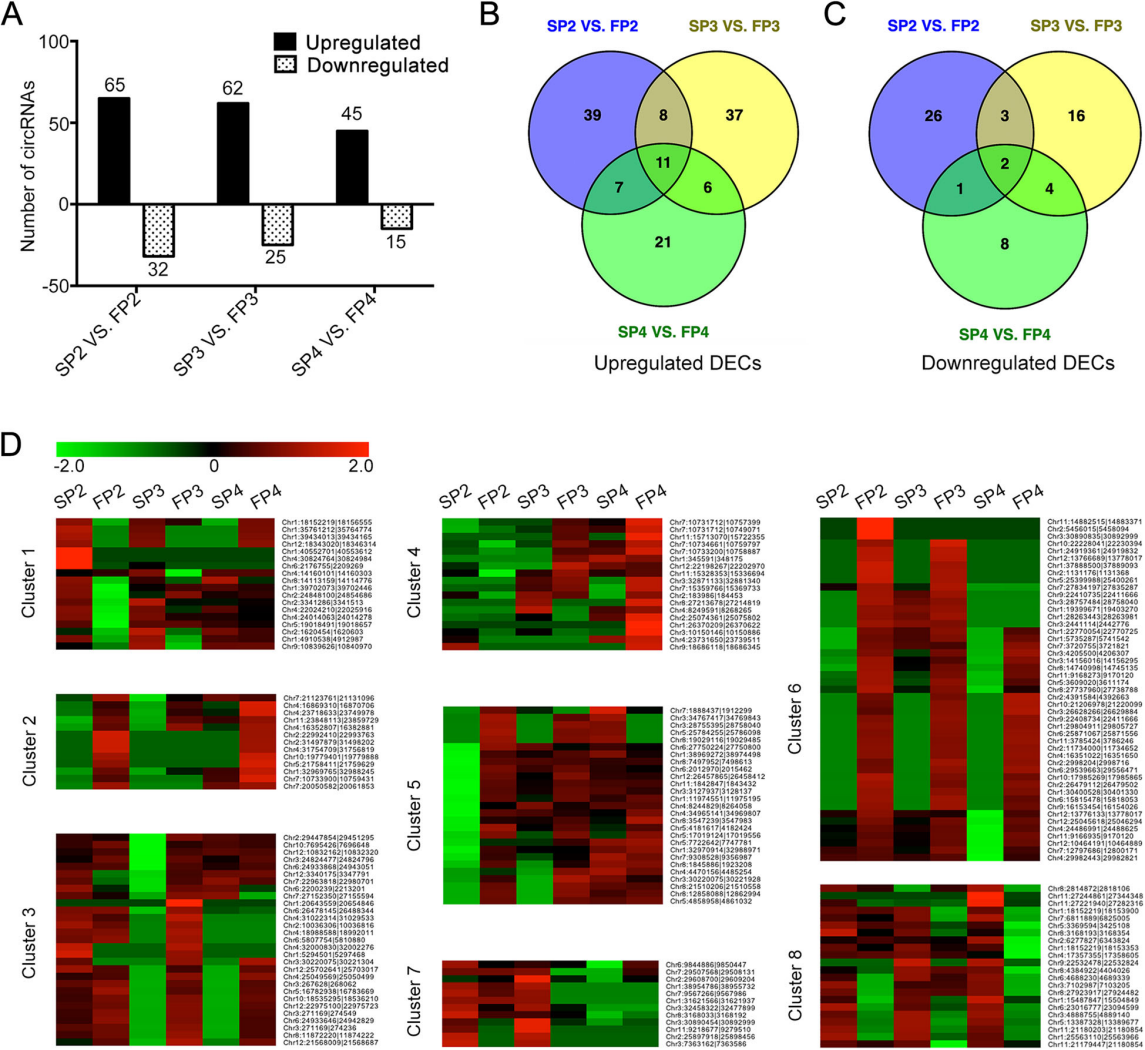
Fertility-specific expression of circRNAs **a** Detailed statistics of differentially expressed circRNAs (DECs) between WXS (S) and WXS (F) at P2, P3 and P4 stages. **b** Venn diagrams showing DEGs that are upregulated expressed both in P2, P3 and P4 stages. **c** Venn diagrams showing DEGs that are downregulated expressed both in P2, P3 and P4 stages. **d** Hierarchical clustering of the DECs between WXS (S) and WXS (F).

To identify fertility transition-associated circRNAs in PTGMS rice WXS, we carefully estimated the expression levels of each circRNA based on the normalized transcripts per million (TPM) value of the read counts. We further analyzed the differential expression of the circRNAs between WXS (S) and WXS (F) in three different periods of young panicles, respectively. In total, 186 circRNAs were identified as significantly differentially expressed circRNAs (DECs) (*P* < 0.05, |log_2_(fold change)| ≥ 1) during fertility transition (SP2-FP2, SP3-FP3 or SP4-FP4) (Additional file 4: Table S3). Among them, 97 were differentially expressed between SP2 and FP2, of which 65 were upregulated and 32 were downregulated in FP2. Additionally, 87 circRNAs were found to be differentially expressed at the P3 stage, of which 62 were upregulated and 25 were downregulated in FP3. In addition, 60 DECs were found at the P4 stage, of which 45 were upregulated and 15 were downregulated in FP4 (Fig. 3a). Intriguingly, we found that 13 circRNAs displayed the same trend of significant differential expression in the three stages, of which 11 showed increased expression levels and two showed decreased expression levels in WXS (F) compared with WXS (S) (Fig. 3b, c).

According to the expression patterns of the circRNAs with fertility-specific expression, it might be possible to classify circRNAs. We obtained eight different clusters of the DECs by K-Mean Clustering and Hierarchical Clustering analysis using MeV software (Fig. 3d). The results showed that many circRNAs were highly expressed (F) (Cluster 2, 6), whereas some were expressed at low levels in the young panicles of WXS (F) (Cluster 8). We also found that some circRNAs exhibited specific expression at a single stage: P2 stage (Cluster 5), P3 stage (Cluster 3) or P4 stage (Cluster 4), respectively. Moreover, some circRNAs showed a significant decrease in expression levels both in FP2 and FP3, the two stages of young panicles of WXS (F) (Cluster 1, 7). Remarkably, the highly fertility-specific expression pattern of circRNAs suggested their specific roles in male sterility during the fertility transition of PTGMS rice WXS.

**Fig. 4:**
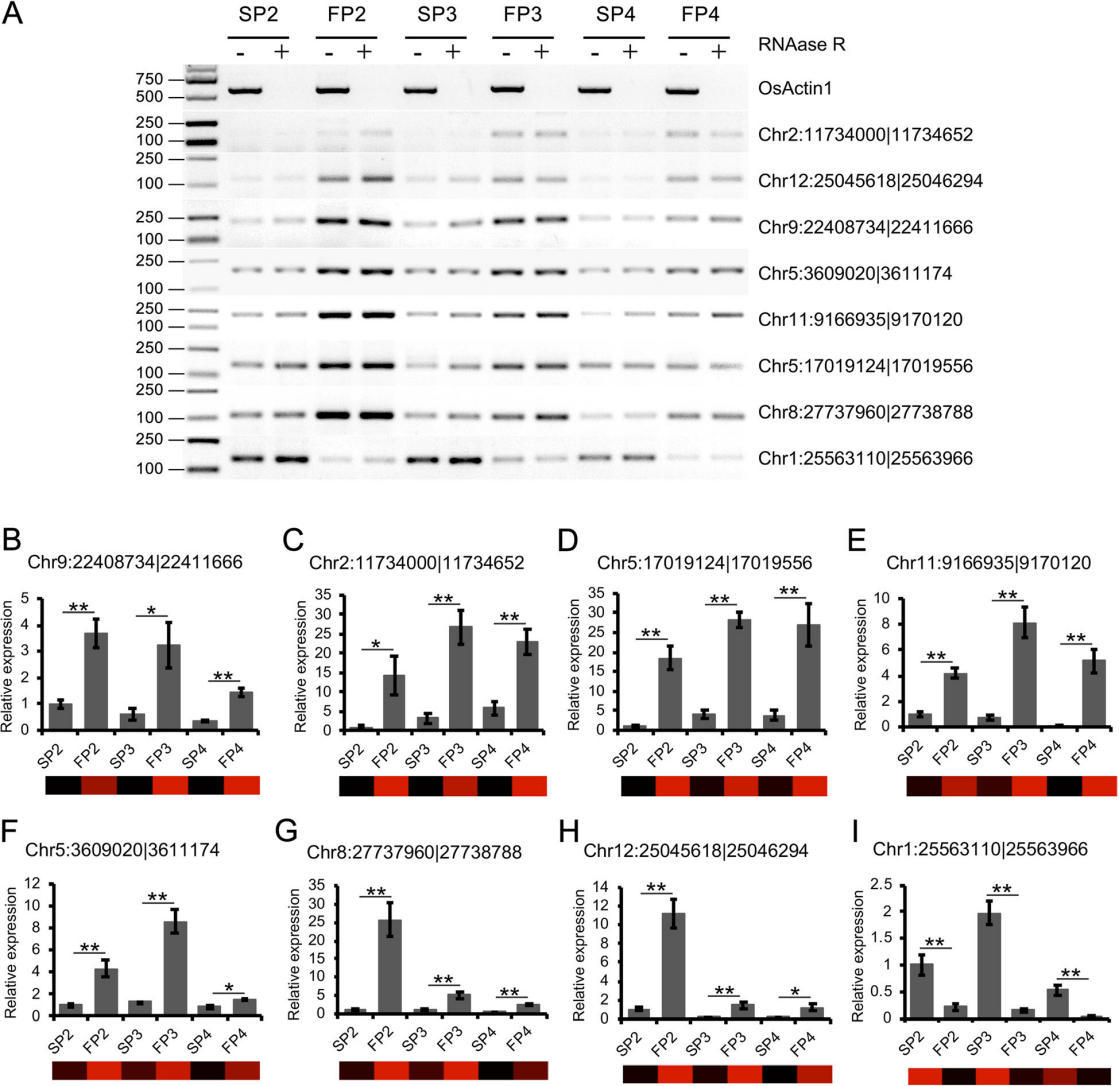
Confirmation of the expression patterns of the DECs by semiquantitative RT-PCR and qRT-PCR **a** Semiquantitative RT-PCR shows that the expression patterns of seven upregulated circRNAs and one downregulated circRNA. *n* = 3 replicates. (**b**-**h**) The expression pattern of seven upregulated circRNAs and (**i**) one downregulated circRNA confirmed by qRT-PCR. A heatmap of each circRNA was generated from the normalized transcripts per million (TPM) value and used to visualize the circRNA expression level in the RNA-sequencing data. *OsActin1* was used as a reference gene. The error bars indicate standard deviation of three replicates. Asterisks indicate a significant difference as determined by Student’s *t*-test (*P < 0.05; **P < 0.01)

To further confirm the expression patterns of the circRNAs with fertility-specific expression, we randomly selected eight DECs, including seven upregulated circRNAs and one downregulated circRNA in the young panicles WXS (F), and we quantified their expression patterns in the RNase R (−) and RNase R (+)-treated RNAs of each sample by semiquantitative RT-PCR (Fig. 4a). Furthermore, qRT-PCR was performed for to validate the expression patterns of the eight DECs. The expression patterns of the eight DECs in the quantitative experiments were highly correlated with the RNA-sequencing results (Fig. 4b - I). This observation indicated that the circRNA expression patterns based on RNA-sequencing data were reliable.

### Functional annotation analysis of parental genes of DECs

**Fig. 5:**
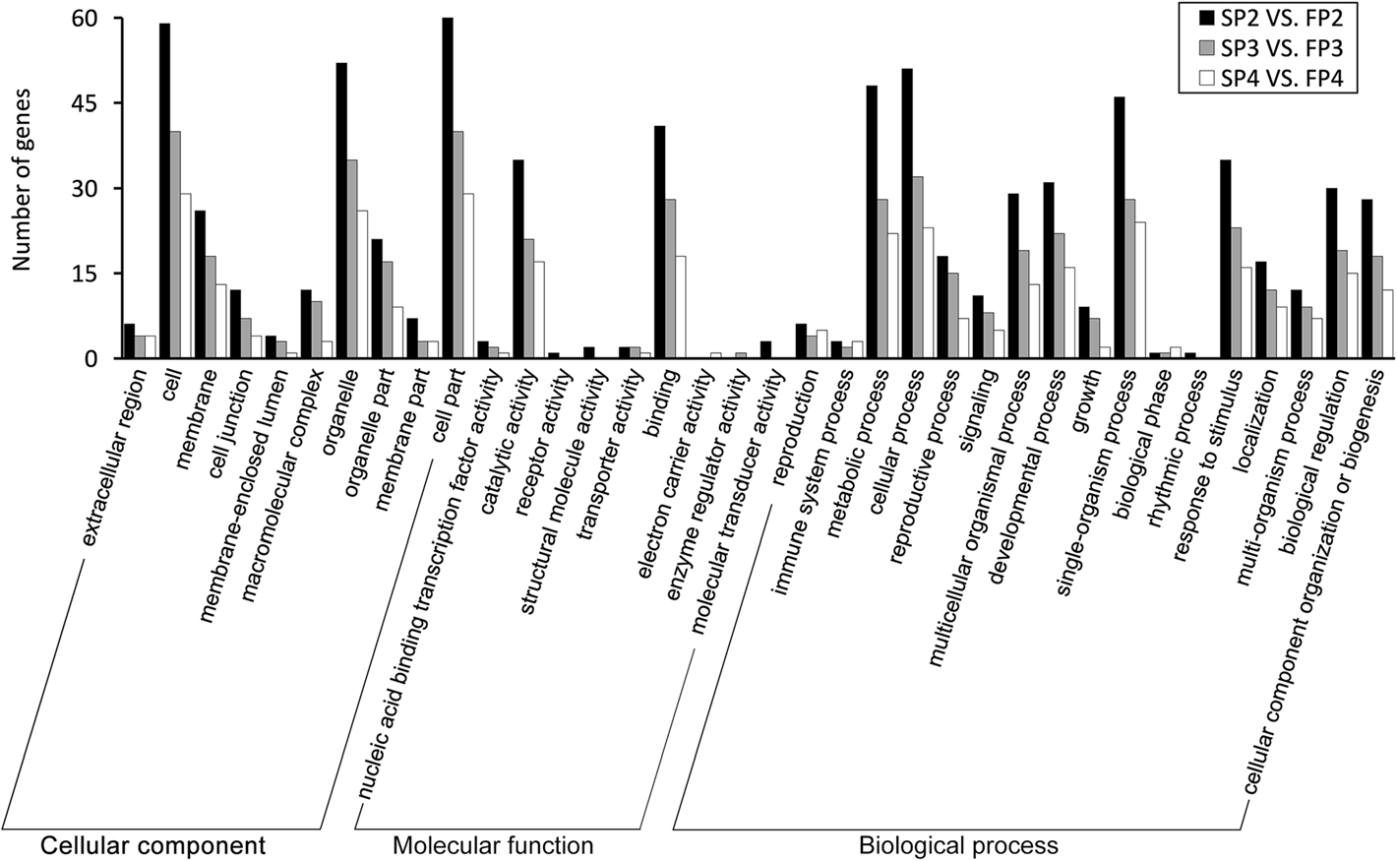
The GO classification of parental genes of DECs at the three developmental stages

To further investigate whether the circRNAs that were significantly differentially expressed during fertility transition have corresponding functions, a functional annotation analysis of 109 parental genes of the DECs was performed (Additional file 5: Table S4). The gene ontology (GO) analysis showed that the parental genes were assigned to three GO categories: biological process, cellular component and molecular function (Fig. 5). In cellular component, the three main categories were cell part, cell and organelle. In molecular function, the most represented categories were catalytic activity and binding. In biological process, some important categories such as metabolic process, biological regulation, development process, response to stimulus, reproduction, and reproductive process were annotated. Furthermore, we detected significant enrichment (*P* < 0.05) of 152, 145 and 117 GO terms at P2, P3 and P4 stages of WXS, respectively (Additional file 6: Table S5). The number of GO terms was clearly more enriched at the P2 and P3 stages than at P4. In addition, the analysis showed that these parental genes of the DECs at the three stages were simultaneously enriched in a variety of biological processes, including cell division (GO:0051301), regulation of cell differentiation (GO:0045595), regulation of hormone levels (GO:0010817), hormone metabolic process (GO:0042445), hormone biosynthetic process (GO:0042446), response to temperature stimulus (GO:0009266), and floral organ development (GO:0048437), which are closely related to the fertility transition. In particular, based on the assigned GO terms at the P3 stage, a significant enrichment of genes involved in reproductive and pollen development (GO:0010228, GO:0009555, GO:0048229, GO:0010208, GO:0048868, GO:0010584) were detected. These results indicated that the parental genes together with the DECs might play a role in the development of anther and pollen by involving various biological processes. KEGG pathway annotation was also performed to further explore the function of the parental genes of the DECs (Additional file 7: Figure S2). A total of 15, 8 and 7 pathways were obtained at the P2, P3 and P4 stages, respectively. KEGG pathway analysis identified the same pathways at three stages, including the biosynthesis of amino acids and ribosome biogenesis in eukaryotes, which were distributed in the two major categories of genetic information processing and metabolism. Specifically, the pathway of plant hormone signal transduction was identified only at the P2 stages; this pathway belongs to the category of environmental information processing.

### Putative functions of rice circRNAs acting as miRNA sponges

CircRNAs have previously been shown to act as targets of miRNAs and to regulate the corresponding miRNA target genes by the competing endogenous RNA (ceRNA) networks, also known as miRNA sponges [20]. Hence, we first predicted the potential of all the circRNA candidates to act as targets for miRNAs based on sequence complementarity in PTGMS rice WXS. We found that 1704 (17%) circRNAs contained putative miRNA-binding sites, with 66 DECs identified as targets of miRNAs. Among the 1704 circRNAs, a considerable number (56%) contained miRNA-binding sites for one miRNA, followed by 15% with binding sites for two miRNAs. The remaining circRNAs contained several binding sites for three or more miRNAs (Additional file 8: Figure S3).

**Fig. 6:**
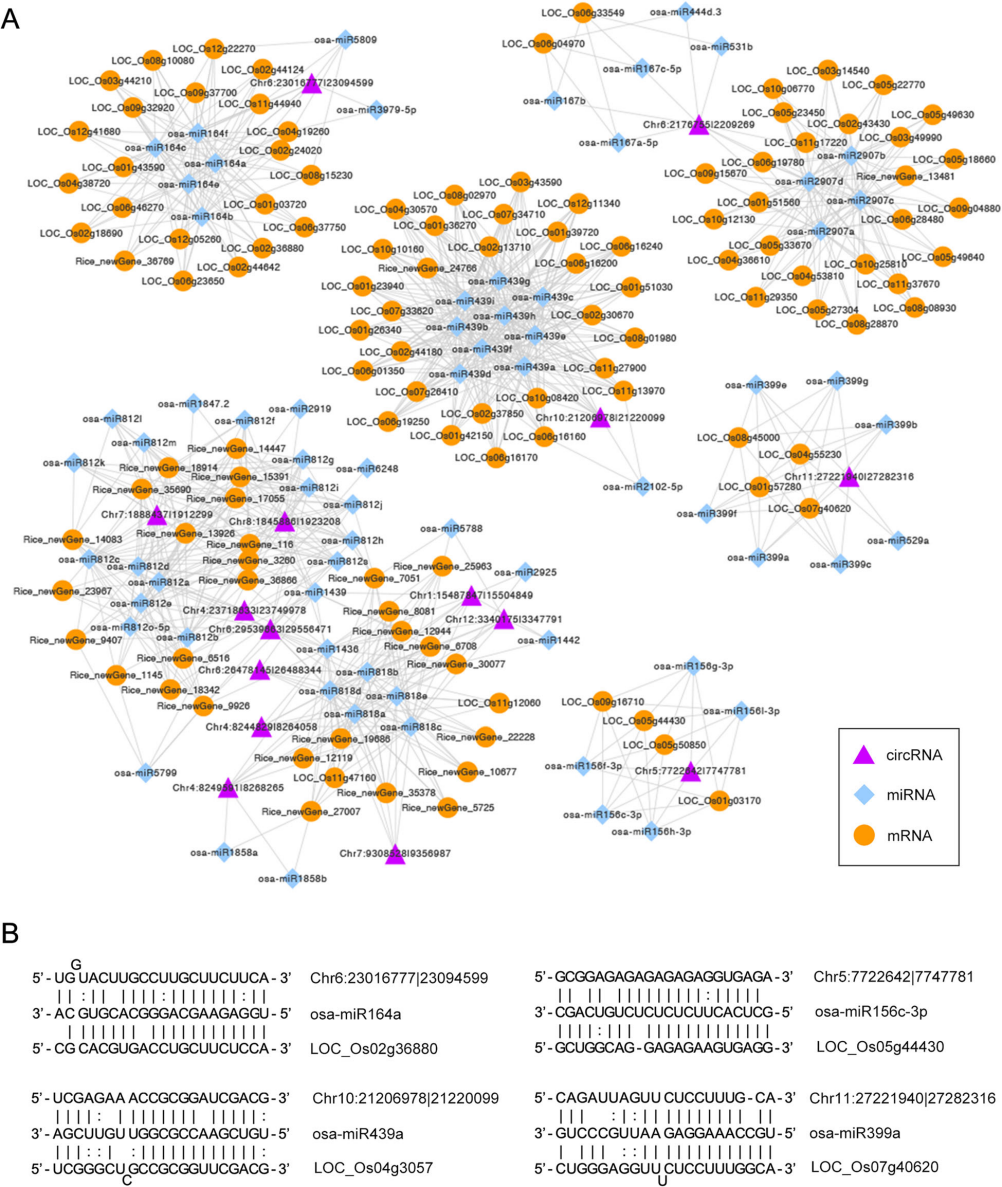
The circRNA-miRNA-mRNA interaction network in PTGMS rice **a** The ceRNA regulatory networks of 15 differentially expressed circRNAs (purple triangle) and miRNA (blue rhombus), as well as their corresponding mRNA targets (orange circle). LOC_Os04g55230, *flo2*; LOC_Os02g36880, *OMTN1*; LOC_Os12g41680, *OMTN3*; LOC_Os06g46270, *OMTN4*; LOC_Os06g23650, *OMTN5*; LOC_Os08g10080, *OMTN6*; LOC_Os03g49990, *OsGAI*; LOC_Os01g43590, *OsHsfC1a*; LOC_Os04g38720, *OsNAC2*; LOC_Os02g37850, *OsOSD1*; LOC_Os09g15670, *OsPP108*; LOC_Os08g45000, *OsPT6*. **b**The binding sites of circRNA, miRNA and the corresponding mRNA.

In the present study, we further identified the miRNAs targeting mRNAs in our transcriptome sequencing using Target Finder software. It is well-known that the potency of a ceRNA is determined by the number of miRNAs; that is, the circRNA and miRNA target gene in a sturdy ceRNA network will compete for multiple miRNAs [57]. Hence, we screened the candidate ceRNA pairs, given that the number of identical miRNAs between the circRNAs and mRNAs was no less than three. Eventually, a total of 15 DECs were identified as potential miRNA sponges, which regulated the expression of target genes through ceRNA networks (Additional file 9: Table S6). The circRNA-miRNA-mRNA networks in their entirety were delineated by Cytoscape software (Fig. 6a). Furthermore, illustrations of the four circRNAs are provided in Fig. 6b to display the interaction of the circRNA, miRNA and the corresponding target mRNA. Among the 15 DECs, 102 target genes were identified and participate in metabolic process, development process, response to stimulus, reproductive process, and other multiple biological processes associated with fertility transition of pollen, as indicated by GO enrichment analysis (Additional file 10: Figure S4). Therefore, circRNAs might play roles in the fertility transition by interacting with miRNAs in the PTGMS rice line.

## Discussion

At present, circRNAs have attracted the attention of an increasing number of researchers. Tens of thousands of circRNAs have been found in various eukaryotic species, and this number is rapidly increasing [58, 59]. A total of 2354 circRNAs have recently been identified in leaves and panicles of rice, certain rice circRNAs have tissue-specific expression, and the overexpression of a circRNA could greatly reduce the expression level of its parental gene [17]. Moreover,12,037 rice circRNAs have been identified in roots, some of which are detected in response to phosphate starvation [16]. In addition, nearly 3000 circRNAs with full-length sequences have been identified in rice, most of which are flanked by the diverse non-GT/AG splicing signals [31]. These results suggest that circRNAs are widely present in rice, while extremely little is known regarding the biological roles of circRNAs in the fertility transition of the two-line rice. Here we profile the circRNAs from 18 ssRNA-seq libraries, which are RNA samples from the young panicles of the WXS fertile rice line and sterile rice line at three developmental stages. Our findings clearly revealed that circRNAs might be endogenous noncoding regulators of flower and pollen development in the PTGMS rice line.

In the present study, a total of 9994 circRNAs were detected in WXS, of which 5305 were known circRNAs and 4689 were novel in rice, and deriving from diverse genomic regions. Among these circRNAs, the majority belonged to the exonic circRNAs generated from the exons of protein-coding genes, and the remaining small number of circRNAs were intronic and intergenic circRNAs. This result is consistent with previous studies, in which most circRNAs were exonic in rice and Arabidopsis [16, 17]. Moreover, a very large percentage of circRNAs were shorter than 1 kb in length, while the mean length of all cirRNAs included the intronic, the intergenic and the exonic was 1603 bp. The mean length of exonic circRNAs has been reported to be only 474 bp in rice [17]. Thus, we conclude that the sequence length of exonic circRNA is generally short, while that of the intronic and the intergenic circRNA is particularly long in rice. Additionally, we systematic analyzed other characteristics of the rice circRNAs. Our results showed that a large number of circRNAs were expressed at an extremely low levels in rice. Simultaneously, we found that circRNAs were derived more frequently from chromosome 1 than others. In addition, it should be noted that most parental genes produce only one circRNA, although some genes produce more than one circRNA, which is consistent with previous reports in plants [16]. These results may be the common features of circRNAs in rice.

The fertility transition of the PTGMS rice line is a complex process that involves flowering, pollen development and other reproductive processes. The sterility-fertility transition is controlled by a cross-talk between certain genetic networks and environmental conditions (such as photoperiod and temperature) during young panicle development [51, 52]. To date, several genes that control PTGMS in different rice line have been identified such as *ptgms2–1* [60], *pms1(t)* [61] and *p/tms12–1* [54]. It has been reported that *p/tms12–1* encodes a noncoding RNA precursor of a 21-nt small RNA, which is an important regulator of male sterility-fertility transition in the PTGMS rice lines. In contrast, the roles of circRNAs in the fertility transition of PTGMS has not been reported. Recently, several studies of circRNAs in several plants have revealed that most of them exhibit differential expression patterns in response to abiotic and biotic stress as well as at different developmental stages [32, 34, 35, 40, 42, 62]. In the present study, among the 9994 identified circRNAs, 186 circRNAs were significantly differentially expressed in the fertile line rice compared with the sterile line rice at the P2, P3 or P4 developmental stages (Fig. 3). These fertility-specific expression patterns of circRNAs indicated that they might respond to photoperiod and temperature stimulation and regulate the fertility transition of pollen in the PTGMS line. Moreover, 11 and two circRNAs that were up-regulated or down-regulated both in three different stages were credible regulators, while the regulatory mechanisms must be further investigated. Interesting, it has been reported that pollen abortion in PGMS rice is initiated already at the stage of PMC formation and continues throughout the whole process of pollen development, which is closely associated with abnormal degeneration of the tapetum [63]. It has also been reported that a large number of microspore mother cells (MMC) in TGMS rice undergo abnormal meiosis under high-temperature conditions, leading to male sterility [55]. Based on our data, the number of DECs at the P2 and P3 stages was larger than that at the P4 stage, indicating that WXS rice was more sensitive to environmental conditions in the PMC formation stage and meiosis stage than that in the microspore formation stage, although both are critical periods for pollen development in PTGMS rice.

Previous research has indicated that some circRNAs participate in regulating the expression of their parental genes in humans, animals and plants. For instance, some intronic circRNAs have an abundance in the nucleus and promote transcription of their parental gene by interacting with the polymerase II (Pol II) machinery [7]. Other intergenic circRNAs predominantly localize in the nucleus, enhancing transcription of their parental genes via interacting with U1 small nuclear ribonucleoprotein (snRNP) [22]. Furthermore, some circRNAs have conserved functions in regulating the expression of parental genes by competing with canonical splicing [23]. In plants, the expression patterns of some circRNAs have a significant positive correlation with their parental genes [16, 30, 62]. Nevertheless, overexpression of plant circRNAs could reduce the expression level of their parental genes [17, 32], suggesting that circRNAs might also function as negative regulators of their parental genes. Recently, an Arabidopsis circRNA derived from the *SEPALLATA*3 gene has been reported to negatively regulate the splicing of the cognate mRNA through R-loop formation [43]. In our study, to further understand the roles of circRNAs in PTGMS rice, GO analysis and KEGG pathway analysis were performed for the parental genes of the DECs in WXS (S) and WXS (F) at the P2, P3 and P4 stages. GO analysis showed that the parental genes were involved in different biological processes, cellular components, and molecular functions (Fig. 5). Based on the assigned GO terms, some parental genes were classified into the categories of metabolic process, biological regulation, development process, response to stimulus, reproduction, and reproductive process, suggesting that circRNAs may contribute distinctly during the fertility transition by associating with different biological processes. Notably, the GO terms related to rice fertility transition, including the regulation of cell differentiation (GO:0045595), response to temperature stimulus (GO:0009266), and floral organ development (GO:0048437), were commonly enriched, suggesting the involvement of circRNAs in regulating the fertility transition. Moreover, regulation of the hormone levels (GO:0010817), hormone metabolic process (GO:0042445), and hormone biosynthetic process (GO:0042446) were observed during GO enrichment. It has been reported that the two plant hormones auxin and jasmonic acid play pivotal roles in male sterility and pollen development [64, 65]. In addition, pollen development (GO:0009555), pollen wall assembly (GO:0010208), pollen tube development (GO:0048868), and pollen exine formation (GO:0010584) were specifically enriched in the P3 stage, suggesting the PMC meiosis stages is the key period of the fertility transition of pollen in WXS rice. We also found an enrichment of this GO term at the P2 and P3 stages to a greater extent than at the P4 stage, suggesting that the process of fertility transition is more complex during the PMC formation and meiosis stages. Additionally, KEGG pathway analysis was used to further predict the function of parental genes of DECs. The KEGG pathways obtained in the three stages were mainly associated with genetic information processing and metabolism. Compared with the P3 and P4 stages, one parental gene (OsRR1) was involved in the plant hormone signal transduction pathway at the P2 stage. These results revealed the parental genes of DECs in the fertility transition involved in development, stimulation-response, hormonal regulation and reproduction, implying that circRNAs may play important roles in the fertility transition of PTGMS rice by the metabolic pathway and hormone signaling pathway.

Recent evidence in human and animals has confirmed that circRNAs may act as potential miRNA sponges, which sequester miRNA away from its mRNA target through circRNA-miRNA-mRNA networks. For example, a human circRNA *ciRS-7/CDR1* harboring more than 70 binding sites of miR-7, and a mouse circRNA *Sry* harboring 16 binding sites of miR138, can function as efficient microRNA sponges [2, 20]. Additionally, a circRNA *circHIPK3* could act as a sponge for 9 miRNAs with 18 potential binding sites in human cells [21]. In plants, however, many circRNAs acting as miRNA sponges have been predicted, yet no direct experimental evidence has been provided [30, 34, 41]. It is reported that 235 circRNAs have putative miRNA-binding sites, and only 31 circRNAs have two or more miRNA-binding sites in rice [17]. In the present study, 1704 circRNAs had putative miRNA-binding sites, and the number of miRNA-binding sites of these circRNAs greatly varied. Furthermore, we found that 15 DECs functioned as miRNA sponges that regulate the expression of mRNAs through ceRNA networks in the fertility transition of WXS rice (Fig. 6a). Among these 15 circRNAs, each circRNA was the target of several miRNAs, which could target multiple mRNAs to form the sturdy ceRNAs networks similar to that in humans and animals. This result implied that circRNAs might play some roles in the fertility transition by interacting with miRNAs. For instance, circRNA Chr6:23016777|23,094,599 and Chr5:7722642|7,747,781 were identified as target mimics for the osa-miR164 and osa-miR156 families, respectively. These two miRNA families and their target genes were found to be involved in the fertility transition of WXS rice in our previous study [56]. Moreover, Chr10:21206978|21,220,099 and Chr4:23718633|23,749,978 were significantly upregulated both in three different stages of WXS (F), identified as the miRNA sponge for osa-miR439 and osa-miR812 in the study, suggesting that these circRNAs might play a crucial role in regulating the fertility transition of PTGMS rice. Another circRNA, Chr11:27221940|27,282,316, can act as a miRNA decoy of miR399, which is an ambient temperature-responsive flowering regulator [66]. Moreover, by interacting with miRNA, circRNAs could regulated the expression of target genes which were required for metabolic process, development process, response to stimulus, reproductive process, and other multiple biological processes associated with fertility transition of pollen. Above all, some circRNAs can act as miRNA sponges and may be able to influence the expression of many important genes that regulate the fertility transition in PTGMS rice. Nevertheless, their authenticity requires further experimental validation. In contrast, we found that a total of 66 DECs had miRNA binding sites, 15 of which were identified as miRNA sponges, indicating that the interaction between circRNA and miRNA is due not only to the ability of the circRNAs to function as microRNA sponges, but also the expression levels of circRNAs might be regulated by the miRNAs through miRNA-mediated target circRNA cleavage. Of course, further function investigates of circRNAs in PTGMS rice are required to fully elucidate the regulatory mechanism of circRNA on fertility transition.

## Conclusions

In the present study, a total of 9994 circRNA were obtained in WXS rice, of which 186 were differentially expressed between WXS (F) and WXS (S) at three development stages of young panicles. Functional annotation of the parental genes of DECs revealed that circRNAs may play roles in the fertility transition of PTGMS rice by the metabolic pathway and hormone signaling pathway. Moreover, we have constructed the putative circRNA-mediated ceRNA networks in PTGMS rice related to fertility transition. In summary, we investigated the abundance and characteristics of circRNAs, and we also explored the expression patterns and functions of circRNAs in the PTGMS rice line WXS. Our results indicated that rice circRNAs might have important biological roles, and might be essential regulators of the fertility of pollen, and shed new light on the regulatory mechanism of the fertility transition in the PTGMS rice line.

## Methods

### Plant materials and growth conditions

The PTGMS rice line, WXS, was used as an experimental material for RNA-seq, which was selected and bred by our laboratory, and obtained the new plant variety right (CNA20120607.9) granted by the Ministry of Agriculture of the People’s Republic of China. The seeds were obtained from State Key Laboratory of Hybrid Rice, College of Life Sciences, Wuhan University, Wuhan, China. During summer in 2017, WXS rice was grown in the experimental field of Wuhan University (30°54′ N, 114°37′ E), Wuhan, Hubei province of China. Under natural conditions, with daily average temperatures that were higher than 24 °C and average day lengths that were longer than 12 h, WXS could produce male-sterility rice (WXS (S)) that did not form pollen (Additional file 11: Figure S5). When the panicle length was approximately 1 cm, some of the rice plants were transferred into a fully intelligent artificial climate plant incubator for a short period of sunshine treatment under short lighting (12 h light/12 h dark) at low day average temperatures (approximately 21 °C) for 3–4 weeks. These WXS plants treated with short sunshine and low temperatures would become male-fertile rice (WXS (F)), with a normal anther morphology (Additional file 11: Figure S5). The young panicles of WXS (S) and WXS (F) were harvested upon formation of the pollen mother cell (PMC) (P2), meiosis of the PMC (P3) and formation of the microspore (P4) stages, and correspondingly designated as SP2, SP3, SP4, FP2, FP3, and FP4, respectively. Immediately upon harvesting, the panicles were frozen in liquid nitrogen and stored at − 80 °C.

### RNA extraction, library construction, and RNA sequencing

A total of 18 strand-specific RNA libraries (SP2–1, SP2–2, SP2–3, FP2–1, FP2–2, FP2–3, SP3–1, SP3–2, SP3–3, FP3–1, FP3–2, FP3–3, SP4–1, SP4–2, SP4–3, FP4–1, FP4–2, and FP4–3) were constructed from the young panicles of WXS (S) and WXS (F) at the P2, P3 and P4 stages for RNA sequencing. Three separate libraries for each condition were used. Total RNA was extracted using TRIzol reagent (TaKaRa, Dalian, China) according to the manufacturer’s protocols to construct sequencing libraries. Subsequently, RNA degradation, contamination, and DNA contamination were monitored on 1.5% agarose gels. Then, the RNA concentration and purity were checked using a NanoDrop 2000 Spectrophotometer (Thermo Fisher Scientific, Wilmington, DE). The RNA integrity was assessed using the RNA Nano 6000 Assay Kit of the Agilent Bioanalyzer 2100 System (Agilent Technologies, CA, USA).

After quality confirmation of the RNA, 1.5 μg RNA per sample was treated with the Ribo-Zero rRNA Removal Kit (Epicentre, Madison, WI, USA) to remove ribosomal RNA (rRNA). Then, according to the manufacturer’s recommendations, sequencing libraries were constructed using the NEBNextR UltraTM Directional RNA Library Prep Kit for IlluminaR (NEB, USA), and they were purified with AMPure XP Beads (Beckman Coulter, Beverly, USA) for selective insertion of 150–200 bp. Next, the library quality was assessed on the Agilent Bioanalyzer 2100. Clustering of the samples was then performed on a cBot Cluster Generation System using TruSeq PE Cluster Kitv3-cBot-HS (Illumina) following the manufacturer’s instructions. Finally, the library preparations were sequenced on the Illumina HiSeq Xten platform. The libraries were constructed and sequenced by Biomarker Biotechnology Corporation (Beijing, China).

### Identification of circRNAs

After RNA sequencing, the raw data (raw reads) were first processed using custom Perl scripts. In this step, clean data (clean reads) were obtained by removing reads containing adapter, poly-N and low-quality raw data. Then, the Q20, Q30, GC-content and sequence duplication level of the clean data were calculated. All the clean data with high quality were used for the identification of circRNAs according to the methods of Ye et al. [16]. Clean reads were mapped to the rice reference genome based on MSU-v7.0 (http://rice.plantbiology.msu.edu/pub/data/Eukaryotic_Projects/o_sativa/annotation_dbs/pseudomolecules/version_7.0/) using the software of HISAT2 (version 2.0.5). The reads that could not be mapped to the genomes were obtained. For these unmapped reads, the 20-nt anchors were first extracted from both ends and aligned independently to the rice reference genomes to identify the unique anchor positions by a widely using tool find_circ as described previously [2]. The reversed orientation of the aligned anchors suggested circRNA splicing. Then, the anchor alignments were extended to generate the GU/AG splice sites flanking the complete read alignments and breakpoints. Finally, a candidate circRNA was identified if it had at least two distinct back-spliced reads.

### Validation of circRNAs

The circRNA candidates were validated using the following procedure. First, the remaining total RNAs from the RNA sequencing were used to synthesize cDNAs with random primers using the RevertAid First Strand cDNA Synthesis Kit (Fermentas, USA) following the manufacturer’s instructions. Secondly, the genomic DNA (gDNA) was extracted from WXS rice using the conventional cetyltrimethylammonium bromide (CTAB) method. The genomic DNA was used as a negative control for divergent primers. Third, divergent and convergent primers were designed according to the methods described in previous studies [2, 16, 17]. Convergent primers were used as a positive control. All primers are listed in the Additional file 12: Table S7. Finally, polymerase chain reaction (PCR) was performed with T3 DNA polymerase (Tsingke, Beijing, China) according to the manufacturer’s instructions to amplify back-spliced junction sites of circRNAs. The PCR products were separated using 1% agarose gel with the Super GelRed (US Everbright Inc), and the bands were excised and purified. Sanger sequencing were carried out to further confirm the junction reads.

### Semiquantitative and quantitative real-time PCR

After total RNA was extracted from young panicles of WXS (S) and WXS (F) at three developmental stages, 2 μg of total RNA from each sample was incubated for 20 min at 37 °C with or without RNase R (Epicentre Technologies, Madison, WI). Subsequently, RNase R-treated RNAs and untreated RNAs were retro-transcribed to synthesize cDNA with random primers using the RevertAid First Strand cDNA Synthesis Kit (Fermentas, USA). For the internal standard OsActin1, cDNA templates were reverse-transcribed using Oligo(dT)18 primers. In particular, divergent primers annealing to circRNA was used to determine the circRNA abundance. All cDNAs were diluted 10 times. For semiquantitative PCR, divergent PCR products were amplified for 35 cycles using T3 DNA polymerase (Tsingke, Beijing, China) according to the manufacturer’s protocols, while OsActin1 products were amplified for 27 cycles. For quantitative real-time PCR (qRT-PCR), diluted cDNA was amplified using SYBR-green fluorescence with an ABI StepOnePlus Real-Time PCR System. In this process, 1 μl diluted cDNA was mixed with 5 μl 2 × SYBR reaction mix and 0.2 μM primers in a 10 μl reaction system. The PCR conditions were 30 s at 95 °C followed by 40 cycles of 10 s at 95 °C, 30 s at 56 °C and 15 s at 72 °C. The levels of circRNA transcripts were normalized to the endogenous linear *OsActin1* transcripts. Each set of experiments was repeated three times. The primers used for semiquantitative and quantitative RT-PCR are listed in Additional file 12: Table S7.

### Differential expression analysis

The expression level of circRNA in each sample was calculated based on the junction reads. TPM was used for standardization of the read counts. Differential expression analyses between SP2 and FP2, between SP3 and FP3, and between SP4 and FP4 were performed using the DESeq R package (version 1.10.1). DESeq provides statistical routines for determining differential expression in digital gene expression data using a model based on the negative binomial distribution. The resulting *P* values were adjusted using the Benjamini and Hochberg approach for controlling the false discovery rate. Genes with an adjusted *P* value < 0.01 and an absolute value of log_2_(fold change) ≥ 1 found by DESeq were assigned as differentially expressed. Hierarchical Clustering and K-Means Clustering of the expression patterns were performed using the MutiExperimental Viewer (MeV, version 4.8.1).

### Gene annotation, GO analysis, and KEGG pathway analysis

We annotated the function of parental genes based on the following databases: Pfam (Protein family), KOG/COG (Clusters of Orthologous Groups of proteins), Nr (NCBI nonredundant protein sequences), Swiss-Prot (A manually annotated and reviewed protein sequence database), and KEGG. GO enrichment analysis of the parental genes of DECs was carried out using the topGO R packages (version 2.7). Statistical enrichment of the parental genes of DECs in KEGG pathways was tested using KOBAS software [67].

### CircRNA-associated ceRNA network prediction

To identify circRNAs as miRNA sponges, the sequences of all rice miRNAs were downloaded from miRBase (http://www.mirbase.org), and the sequences of mRNAs were obtained from our transcriptome sequencing in WXS rice. Then, the competing endogenous RNA (ceRNA) networks were predicted using Target Finder software. First, the target circRNAs of rice miRNAs were identified. Second, all target mRNAs of rice miRNAs were identified. Finally, if the circRNA and mRNA shared the same three or more miRNAs, we defined the circRNA as a candidate miRNA sponge of the mRNA gene and thus representative of candidate ceRNA pairs. The ceRNA networks were visualized using Cytoscape (version 3.5.0) to show the potential connections between the circRNA, miRNA, and mRNA.

## Additional files

Additional file 1: **Table S1.** Summary of the deep sequencing and data analysis. (XLSX 11 kb)

Additional file 2: **Table S2.** The known circRNAs in the study identified by the BLASTN. (XLSX 386 kb)

Additional file 3: **Figure S1.** Validation of rice circRNAs candidates. (XLSX 39 kb)

Additional file 4: **Table S3.** The DECs identified in WXS (S) and WXS (F) at the three stages. (1) The differentially expressed circRNAs identified in WXS (S) and WXS (F) at the P2 stage (SP2 VS. FP2). (2) The differentially expressed circRNAs identified in WXS (S) and WXS (F) at the P3 stage (SP3 VS. FP3). (3) The differentially expressed circRNAs identified in WXS (S) and WXS (F) at the P4 stage (SP4 VS. FP4).

Additional file 5: **Table S4.** The annotation of all parental genes of DECs between WXS (S) and WXS (F).

Additional file 6: **Table S5.** The topGO enrichment analysis in parental genes of DECs at the three developmental stages. (1) The biological process of topGO enrichment analysis in parental genes of DECs at the P2 stage. (2) The cellular component of topGO enrichment analysis in parental genes of DECs at the P2 stage. (3) The molecular function of topGO enrichment analysis in parental genes of DECs at the P2 stage. (4) The biological process of topGO enrichment analysis in parental genes of DECs at the P3 stage. (5) The cellular component of topGO enrichment analysis in parental genes of DECs at the P3 stage. (6) The molecular function of topGO enrichment analysis in parental genes of DECs at the P3 stage. (7) The biological process of topGO enrichment analysis in parental genes of DECs at the P4 stage. (8) The cellular component of topGO enrichment analysis in parental genes of DECs at the P4 stage. (9) The molecular function of topGO enrichment analysis in parental genes of DECs at the P4 stage. (XLSX 449 kb)

Additional file 7: **Figure S2.** The KEGG pathway analysis in parental genes of DECs. The KEGG pathway analysis in parental genes of DECs at the P2 stage (A), P3 stage (B), and P4 stage (C), respectively. (TIF 615 kb)

Additional file 8: **Figure S3.** Pie chart showing the number distribution of miRNA targets of circRNAs. (TIF 788 kb)

Additional file 9: **Table S6.** Predicted circRNA-miRNA-mRNA connection for differentially expressed circRNAs in WXS (S) and WXS (F). (XLSX 20 kb)

Additional file 10: **Figure S4.** The GO classification of the target genes in the ceRNA networks. (TIF 1637 kb)

Additional file 11: **Figure S5.** Anther morphology and pollen fertility of WXS. Normal anthers (A) in WXS (F) and abnormal anthers (B) in WXS (S) observed by stereo microscope. Mature pollen (C) in WXS (F) and abortive pollen (D) in WXS (S) stained darkly with 1% potassium iodide solution (I_2_-KI). Scale bars, 1.02 mm (A, B), and 10 μm (C, D). (TIF 2839 kb)

Additional file 12: **Table S7.** The divergent primers for validation of randomly selected circRNAs and qRT-PCR.

## Data Availability

The sequencing data have been deposited in the Sequence Read Archive (SRA) at the National Center for Biotechnology Information (NCBI) under the accession number GSE125608.

## Abbreviations

ceRNA: competing endogenous RNA
circRNA: Circular RNAs
CMS: Cytoplasmic male sterility
DECs: Differentially expressed circRNAs
GO: Gene Ontology
KEGG: Kyoto Encyclopedia of Genes and Genomes
MIMV: Maize Iranian mosaic virus
MMC: Microspore mother cells
P2: The PMC formation stage
P3: The meiosis stage
P4: The microspore formation stage
PCR: Polymerase chain reaction
PGMS: Photoperiod-sensitive genic male sterile
PMC: Pollen mother cell
PTGMS: Photo-thermosensitive genic male sterile
qRT-PCR: real-time quantitative RT-PCR
RNase R (−): without RNase R-treatment
RNase R (+): with RNase R-treatment
RT-PCR: Reverse transcription (RT)-PCR
SNP: Single nucleotide polymorphism
TGMS: Thermosensitive genic male sterile
TPM: Transcripts per million
WXS (F): Wuxiang S fertile line rice
WXS (S): Wuxiang S sterile line rice
WXS: Wuxiang S

## References

[1] Jeck WR, Sorrentino JA, Wang K, Slevin MK, Burd CE, Liu JZ (2013). Circular RNAs are abundant, conserved, and associated with ALU repeats. RNA.

[2] Memczak S, Jens M, Elefsinioti A, Torti F, Krueger J, Rybak A (2013). Circular RNAs are a large class of animal RNAs with regulatory potency. Nature.

[3] Zhang Y, Xue W, Li X, Zhang J, Chen S, Zhang JL (2016). The biogenesis of nascent circular RNAs. Cell Rep.

[4] Gao Y, Wang J, Zheng Y, Zhang JY, Chen S, Zhao FQ (2016). Comprehensive identification of internal structure and alternative splicing events in circular RNAs. Nat Commun.

[5] Zhang XO, Dong R, Zhang Y, Zhang JL, Luo Z, Zhang J (2016). Diverse alternative back-splicing and alternative splicing landscape of circular RNAs. Genome Res.

[6] Zhang XO, Wang HB, Zhang Y, Lu XH, Chen LL, Li YL (2014). Complementary sequence-mediated exon circularization. Cell.

[7] Zhang Y, Zhang XO, Chen T, Xiang JF, Yin QF, Xing YH (2013). Circular intronic long noncoding RNAs. Mol Cell.

[8] Sanger HL, Klotz G, Riesner D, Gross HJ, Kleinschmidt AK (1976). Viroids are single-stranded covalently closed circular RNA molecules existing as highly base-paired rod-like structures. Proc Natl Acad Sci U S A.

[9] Glazar P, Papavasileiou P, Rajewsky N (2014). circBase: a database for circular RNAs. RNA.

[10] Chen X, Han P, Zhou T, Guo XJ, Song XF, Li Y (2016). circRNADb: A comprehensive database for human circular RNAs with protein-coding annotations. Sci Rep.

[11] Liu YC, Li JR, Sun CH, Andrews E, Chao RF, Lin FM (2016). CircNet: a database of circular RNAs derived from transcriptome sequencing data. Nucleic Acids Res.

[12] Chu Q, Zhang X, Zhu X, Liu C, Mao LF, Ye CY (2017). PlantcircBase: a database for plant circular RNAs. Mol Plant.

[13] Salzman J, Gawad C, Wang PL, Lacayo N, Brown PO (2012). Circular RNAs are the predominant transcript isoform from hundreds of human genes in diverse cell types. PLoS One.

[14] Danan M, Schwartz S, Edelheit S, Sorek R (2012). Transcriptome-wide discovery of circular RNAs in archaea. Nucleic Acids Res.

[15] Wang PL, Bao Y, Yee MC, Barrett SP, Hogan GJ, Olsen MN (2014). Circular RNA is expressed across the eukaryotic tree of life. PLoS One.

[16] Ye CY, Chen L, Liu C, Zhu QH, Fan LJ (2015). Widespread noncoding circular RNAs in plants. New Phytol.

[17] Lu TT, Cui LL, Zhou Y, Zhu CR, Fan DL, Gong H (2015). Transcriptome-wide investigation of circular RNAs in rice. RNA.

[18] Moran JV, Salzman J, Chen RE, Wang PL, Brown PO (2013). Cell-type specific features of circular RNA expression. PLoS Genet.

[19] Xia S, Feng J, Lei L, Hu J, Xia LJ, Wang J (2017). Comprehensive characterization of tissue-specific circular RNAs in the human and mouse genomes. Brief Bioinform.

[20] Hansen TB, Jensen TI, Clausen BH, Bramsen JB, Finsen B, Damgaard CK (2013). Natural RNA circles function as efficient microRNA sponges. Nature.

[21] Zheng QP, Bao CY, Guo WJ, Li SY, Chen J, Chen B (2016). Circular RNA profiling reveals an abundant circHIPK3 that regulates cell growth by sponging multiple miRNAs. Nat Commun.

[22] Li ZY, Huang C, Bao C, Chen L, LinM WXL (2015). Exon-intron circular RNAs regulate transcription in the nucleus. Nat. Struct. Mol Biol.

[23] Ashwal-Fluss R, Meyer M, Pamudurti NR, Ivanov A, Bartok O, Hanan M (2014). circRNA biogenesis competes with pre-mRNA splicing. Mol Cell.

[24] Legnini I, Di Timoteo G, Rossi F, Morlando M, Briganti F, Sthandier O (2017). Circ-ZNF609 is a circular RNA that can be translated and functions in myogenesis. Mol Cell.

[25] Yang Y, Fan XJ, Mao MW, Song XW, Wu P, Zhang Y (2017). Extensive translation of circular RNAs driven by N^6^-methyladenosine. Cell Res.

[26] Chen G, Cui J, Wang L, Zhu YF, Lu ZG, Jin B (2017). Genome-wide identification of circular RNAs in *Arabidopsis thaliana*. Front Plant Sci.

[27] Liu T, Zhang L, Chen G, Shi T (2017). Identifying and characterizing the circular RNAs during the lifespan of Arabidopsis leaves. Front Plant Sci.

[28] Dou YC, Li SJ, Yang WL, Liu K, Du Q, Ren GD (2017). Genome-wide discovery of circular RNAs in the leaf and seedling tissues of *Arabidopsis Thaliana*. Curr Genomics.

[29] Sun XY, Wang L, Ding JC, Wang YR, Wang JS, Zhang XY (2016). Integrative analysis of *Arabidopsis thalianat* ranscriptomics reveals intuitive splicing mechanism for circular RNA. FEBS Lett.

[30] Pan T, Sun X, Liu Y, Li H, Deng GB, Lin HH (2018). Heat stress alters genome-wide profiles of circular RNAs in *Arabidopsis*. Plant Mol Biol.

[31] Ye CY, Zhang XC, Chu QJ, Liu C, Yu YY, Jiang WQ (2016). Full-length sequence assembly reveals circular RNAs with diverse non-GT/AG splicing signals in rice. RNA Biol.

[32] Tan JJ, Zhou ZJ, Niu YJ, Sun XY, Deng ZP (2017). Identification and functional characterization of tomato circRNAs derived from genes involved in fruit pigment accumulation. Sci Rep.

[33] Zuo JH, Wang Q, Zhu BZ, Luo YB, Gao LP (2016). Deciphering the roles of circRNAs on chilling injury in tomato. Biochem Biophys Res Commun.

[34] Yin JL, Liu MY, Ma DF, Wu JW, Li SL, Zhu YX (2018). Identification of circular RNAs and their targets during tomato fruit ripening. Postharvest Biol Technol.

[35] Wang JY, Yang YW, Jin LM, Ling XT, Liu TL, Chen TZ (2018). Re-analysis of long non-coding RNAs and prediction of circRNAs reveal their novel roles in susceptible tomato following TYLCV infection. BMC Plant Biol.

[36] Darbani B, Noeparvar S, Borg S (2016). Identification of circular RNAs from the parental genes involved in multiple aspects of cellular metabolism in barley. Front Plant Sci.

[37] Chen L, Zhang P, Fan Y, Lu Q, Li Q, Yan JB (2018). Circular RNAs mediated by transposons are associated with transcriptomic and phenotypic variation in maize. New Phytol.

[38] Tang BH, Hao ZQ, Zhu YF, Zhang H, Li GL (2018). Genome-wide identification and functional analysis of circRNAs in *Zea mays*. PLoS One.

[39] Ghorbani A, Izadpanah K, Peters JR, Dietzgenb RG, Mitter N (2018). Detection and profiling of circular RNAs in uninfected and maize Iranian mosaic virus-infected maize. Plant Sci.

[40] Wang YX, Yang M, Wei SM, Qin FJ, Zhao HJ, Suo B (2016). Identification of Circular RNAs and Their Targets in Leaves of Triticum aestivum L under Dehydration Stress. Front Plant Sci.

[41] Zhao W, Cheng Y, Zhang C, You Q, Shen X, Guo W (2017). Genome-wide identification and characterization of circular RNAs by high throughput sequencing in soybean. Sci Rep.

[42] Chen L, Ding X, Zhang H, He T, Li Y, Wang T (2018). Comparative analysis of circular RNAs between soybean cytoplasmic male-sterile line NJCMS1A and its maintainer NJCMS1B by high-throughput sequencing. BMC Genomics.

[43] Conn VM, Hugouvieux V, Nayak A, Conos SA, Capovilla G, Cildir G, et al. A circRNA from SEPALLATA3 regulates splicing of its cognate mRNA through R-loop formation. Nat plants. 2017;317053. 10.1038/nplants.2017.53 28418376

[44] Cheng J, Zhang Y, Li Z, Wang T, Zhang X, Zheng B (2017). A lariat-derived circular RNA is required for plant development in *Arabidopsis*. Sci China Life Sci.

[45] Zhang Pei, Fan Yuan, Sun Xiaopeng, Chen Lu, Terzaghi William, Bucher Etienne, Li Lin, Dai Mingqiu (2019). A large‐scale circular RNA profiling reveals universal molecular mechanisms responsive to drought stress in maize and Arabidopsis. The Plant Journal.

[46] Itoh J, Nonomura K, Ikeda K, Yamaki S, Inukai Y, Yamagishi H (2005). Rice plant development: from zygote to spikelet. Plant Cell Physiol.

[47] Huang X, Yang S, Gong J, Zhao Q, Feng Q, Zhan Q (2016). Genomic architecture of heterosis for yield traits in rice. Nature..

[48] Cheng SH, Zhuang JY, Fan YY, Du JH, Cao LY (2007). Progress in research and development on hybrid rice: a super-domesticate in China. Ann Bot.

[49] Su N, Hu ML, Wu DX, Wu FQ, Fei GL, Lan Y (2012). Disruption of a rice pentatricopeptide repeat protein causes a seedling-specific albino phenotype and its utilization to enhance seed purity in hybrid rice production. Plant Physiol.

[50] Chang Z, Chen Z, Wang N, Xie G, Lu J, Yan W (2016). Construction of a male sterility system for hybrid rice breeding and seed production using a nuclear male sterility gene. Proc Natl Acad Sci U S A.

[51] Fan Y, Zhang Q (2017). Genetic and molecular characterization of photoperiod and thermo-sensitive male sterility in rice. Plant Reprod.

[52] Li X, He Y, Yang J, Jia YH, Zeng HL (2018). Gene mapping and transcriptome profiling of a practical photo-thermo-sensitive rice male sterile line with seedling-specific green-revertible albino leaf. Plant Sci.

[53] Ding J, Lu Q, Ouyang Y, Mao H, Zhang P, Yao J (2012). A long noncoding RNA regulates photoperiod-sensitive male sterility, an essential component of hybrid rice. Proc Natl Acad Sci U S A.

[54] Zhou H, Liu Q, Li J, Jiang D, Zhou L, Wu P (2012). Photoperiod- and thermo-sensitive genic male sterility in rice are caused by a point mutation in a novel noncoding RNA that produces a small RNA. Cell Res.

[55] Zhou H, Zhou M, Yang Y, Li J, Zhu L, Jiang D (2014). RNase Z^S1^ processes *Ub*_*L40*_ mRNAs and controls thermosensitive genic male sterility in rice. Nat Commun.

[56] Zhang H, Hu J, Qian Q, Chen H, Jin J, Ding Y (2016). Small RNA profiles of the rice PTGMS line Wuxiang S reveal miRNAs involved in fertility transition. Front Plant Sci.

[57] Salmena L, Poliseno L, Tay Y, Kats L, Pandolfi PP (2011). A ceRNA hypothesis: the Rosetta stone of a hidden RNA language?. Cell..

[58] Chen LL (2016). The biogenesis and emerging roles of circular RNAs. Nat Rev Mol Cell Biol.

[59] Holdt LM, Kohlmaier A, Teupser D (2017). Molecular roles and function of circular RNAs in eukaryotic cells. Cell Mol Life Sci.

[60] Xu J, Wang B, Wu Y, Du P, Wang J, Wang M (2011). Fine mapping and candidate gene analysis of ptgms2-1, the photoperiod-thermo-sensitive genic male sterile gene in rice (*Oryza sativa* L.). Theor Appl Genet.

[61] Zhou YF, Zhang XY, Xue QZ (2011). Fine mapping and candidate gene prediction of the photoperiod and thermo-sensitive genic male sterile gene pms1(t) in rice. J Zhejiang Univ Sci B.

[62] Wang Z, Liu Y, Li D, Li L, Zhang Q, Wang S (2017). Identification of circular RNAs in kiwifruit and their species-specific response to bacterial canker pathogen invasion. Front Plant Sci.

[63] Shi Y, Zhao S, Yao J (2009). Premature tapetum degeneration: a major cause of abortive pollen development in photoperiod sensitive genic male sterility in rice. J Integr Plant Biol.

[64] Cheng Y, Dai X, Zhao Y (2006). Auxin biosynthesis by the YUCCA flavin monooxygenases controls the formation of floral organs and vascular tissues in Arabidopsis. Genes Dev.

[65] Ishiguro S, Kawai-Oda A, Ueda J, Nishida I, Okada K (2001). The Defective in anther dehiscience gene encodes a novel phospholipase A1 catalyzing the initial step of jasmonic acid biosynthesis, which synchronizes pollen maturation, anther dehiscence, and flower opening in Arabidopsis. Plant Cell.

[66] Kim W, Ahn HJ, Chiou TJ, Ahn JH (2011). The role of the miR399-*PHO2* module in the regulation of flowering time in response to different ambient temperatures in Arabidopsis thaliana. Mol Cell.

[67] Mao X, Cai T, Olyarchuk JG, Wei L (2005). Automated genome annotation and pathway identification using the KEGG Orthology (KO) as a controlled vocabulary. Bioinformatics..

