# Supplementary figures and images for: Circular RNA profiling of the rice photo-thermosensitive genic male sterile line Wuxiang S reveals circRNA involved in the fertility transition
Source: BMC Plant Biol. 2019 Aug 5;19:340. doi: 10.1186/s12870-019-1944-2 (PMC6683460; doi:10.1186/s12870-019-1944-2)

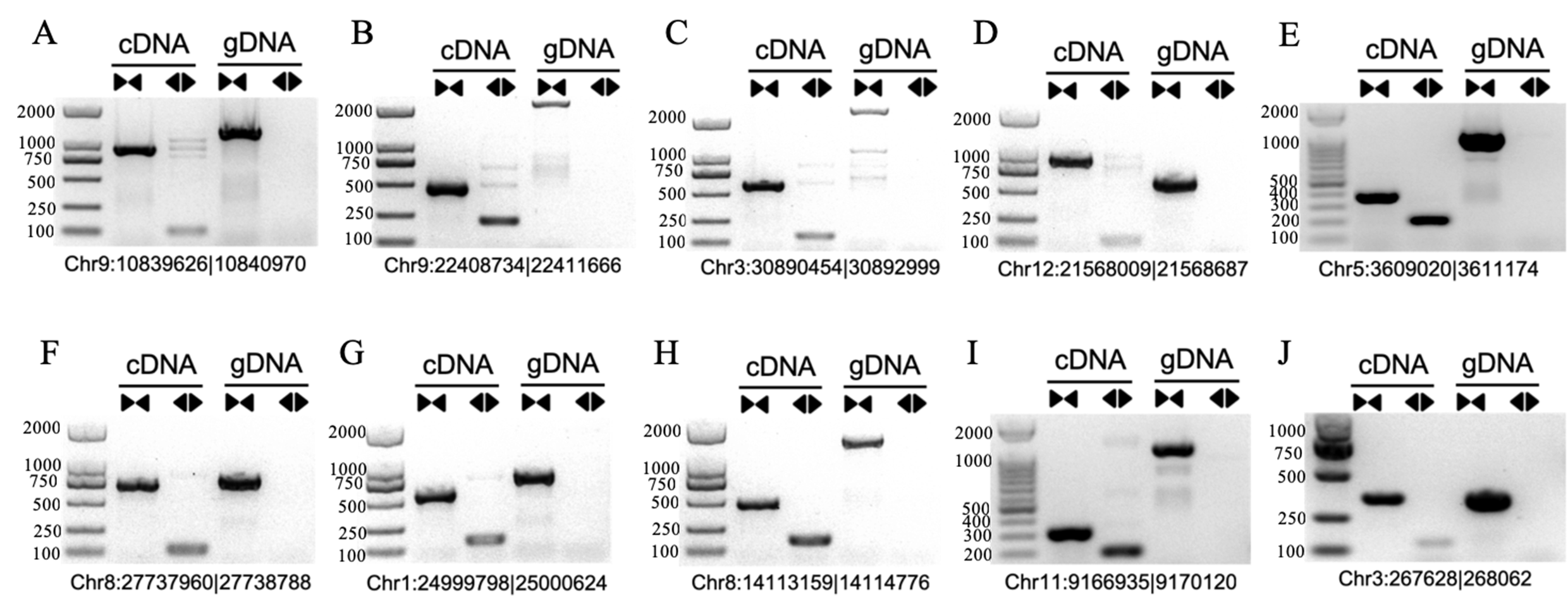

Supplement: Supplementary file 8 — Figure S3. Pie chart showing the number distribution of miRNA targets of circRNAs. (TIF 788 kb) [file 12870_2019_1944_MOESM8_ESM.tif]

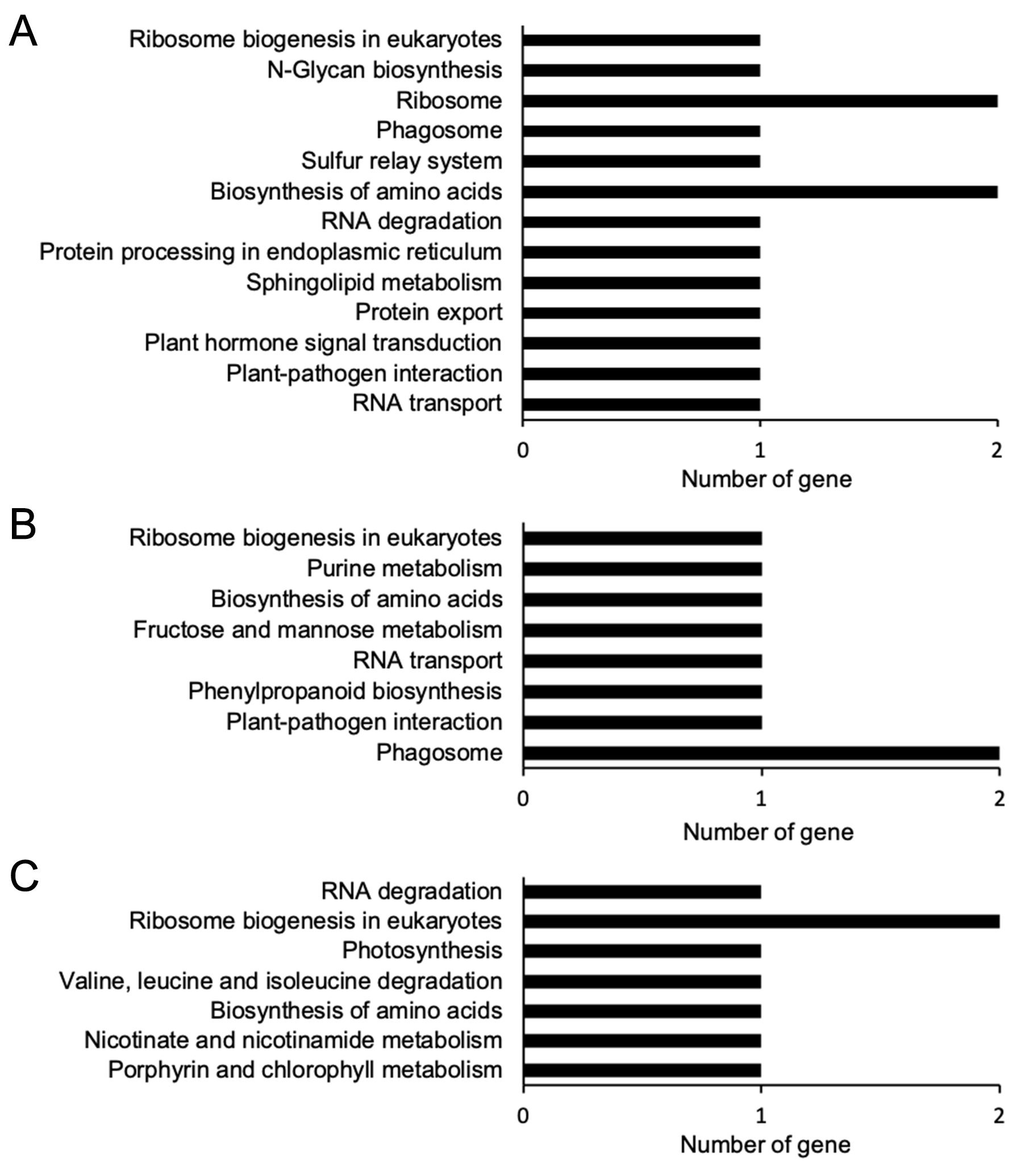

Supplement: Supplementary file 9 — Table S6. Predicted circRNA-miRNA-mRNA connection for differentially expressed circRNAs in WXS (S) and WXS (F). (XLSX 20 kb) [file 12870_2019_1944_MOESM9_ESM.tif]

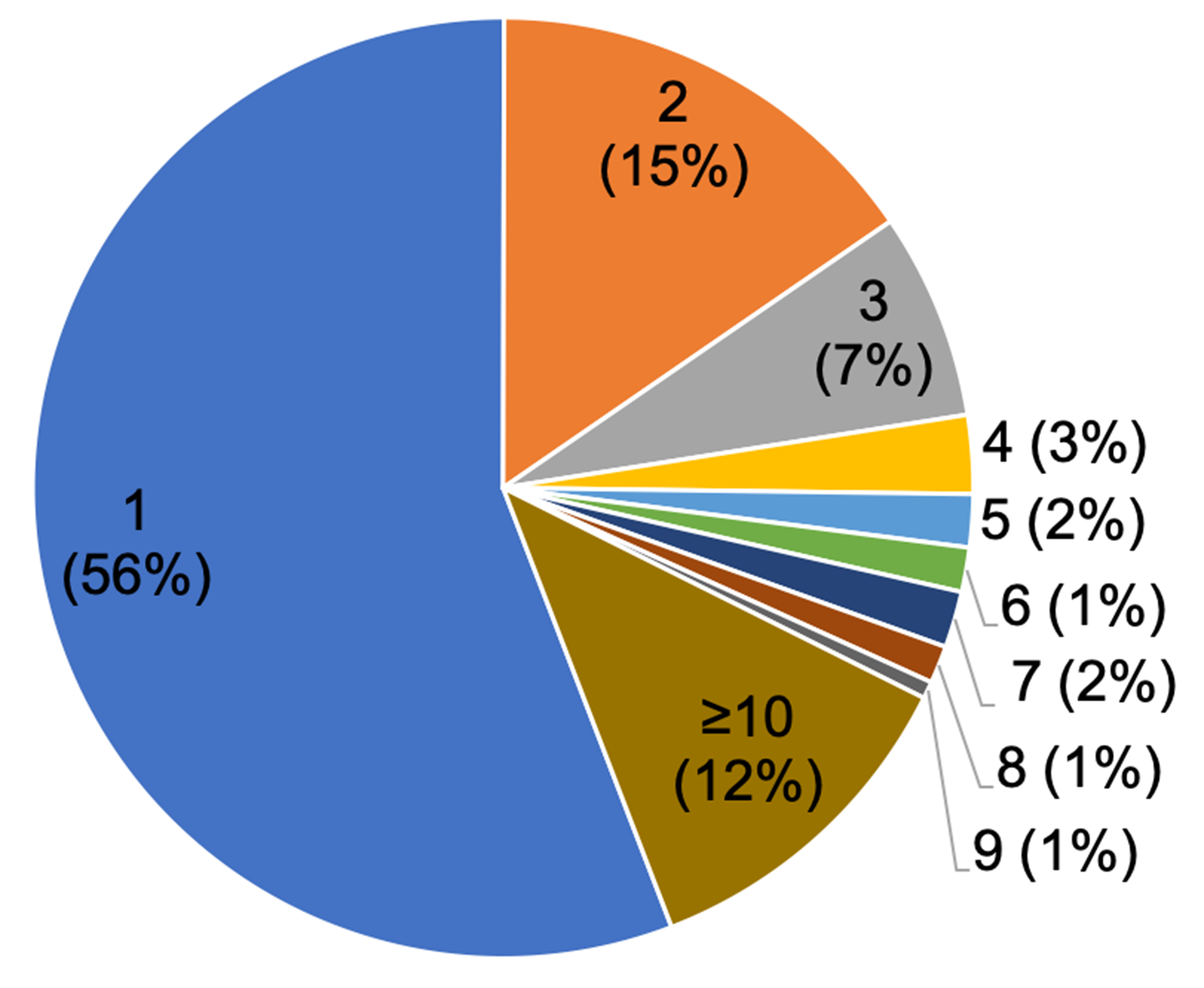

Supplement: Supplementary file 10 — Figure S4. The GO classification of the target genes in the ceRNA networks. (TIF 1637 kb) [file 12870_2019_1944_MOESM10_ESM.tif]

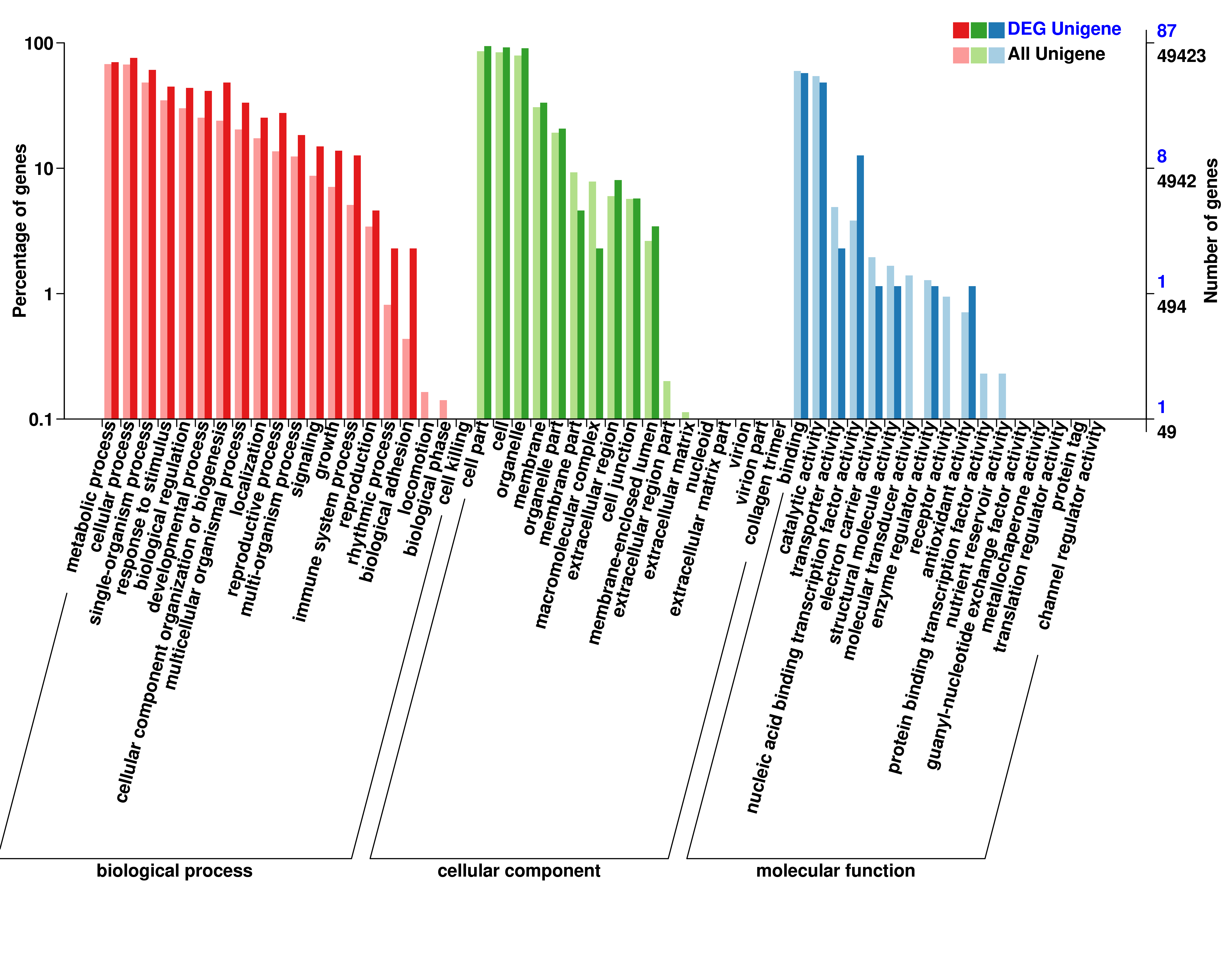

Supplement: Supplementary file 11 — Figure S5. Anther morphology and pollen fertility of WXS. Normal anthers (A) in WXS (F) and abnormal anthers (B) in WXS (S) observed by stereo microscope. Mature pollen (C) in WXS (F) and abortive pollen (D) in WXS (S) stained darkly with 1% potassium iodide solution (I2-KI). Scale bars, 1.02 mm (A, B), and 10 μm (C, D). (TIF 2839 kb) [file 12870_2019_1944_MOESM11_ESM.tif]

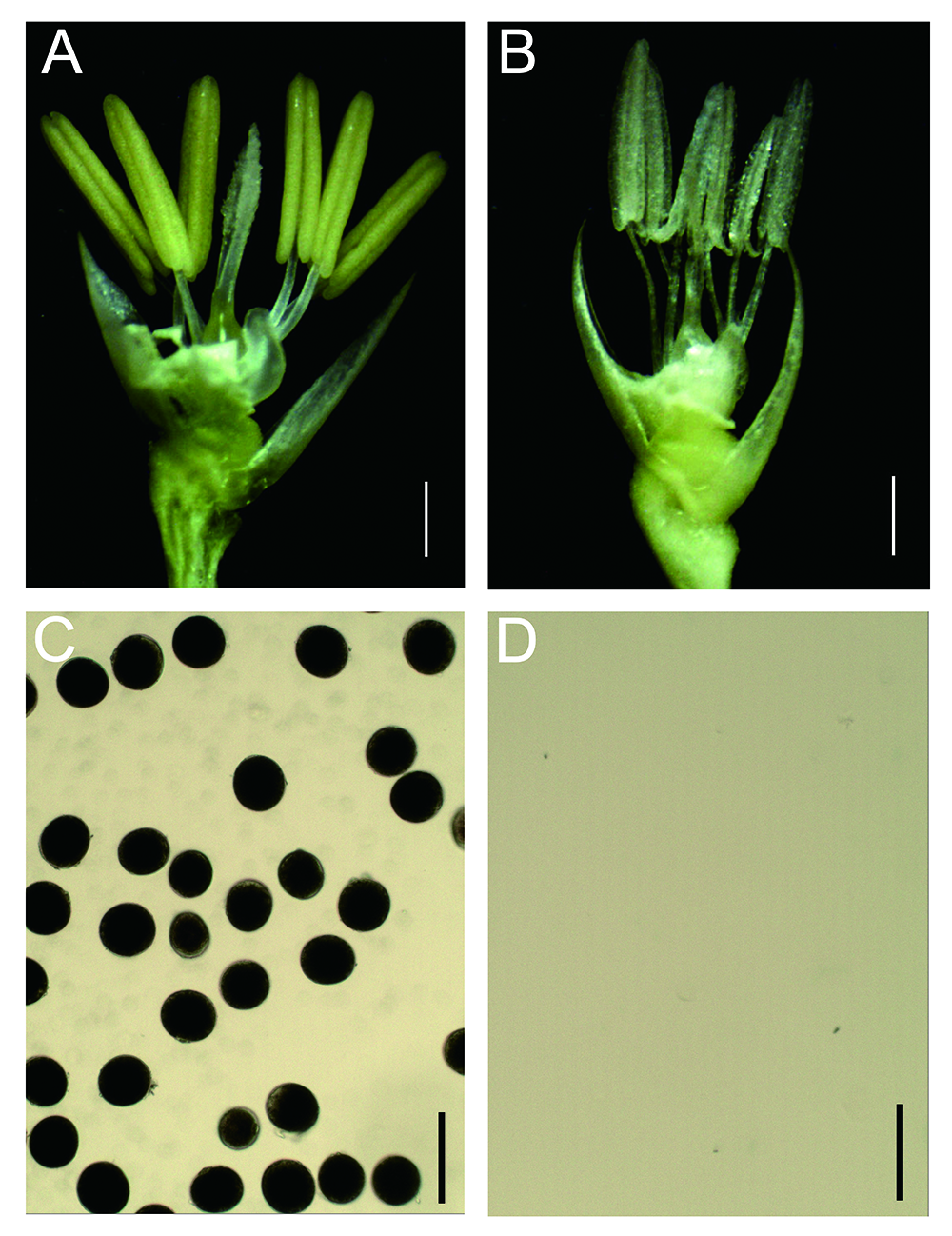

Supplement: Supplementary file 12 — Table S7. The divergent primers for validation of randomly selected circRNAs and qRT-PCR. [file 12870_2019_1944_MOESM12_ESM.tif]
